# Distribution of *Salmonella* spp. Serotypes Isolated from Poultry in Abruzzo and Molise Regions (Italy) during a 6-Year Period

**DOI:** 10.3390/microorganisms10020199

**Published:** 2022-01-18

**Authors:** Margherita Perilli, Silvia Scattolini, Gianluca Ciro Telera, Alessandra Cornacchia, Patrizia Tucci, Flavio Sacchini, Massimo Sericola, Romina Romantini, Francesca Marotta, Andrea Di Provvido, Francesco Pomilio, Fabrizio De Massis

**Affiliations:** 1 Istituto Zooprofilattico Sperimentale dell’Abruzzo e del Molise “G. Caporale”, 64100 Teramo, Italy; m.perilli@izs.it (M.P.); g.telera@izs.it (G.C.T.); a.cornacchia@izs.it (A.C.); p.tucci@izs.it (P.T.); f.sacchini@izs.it (F.S.); m.sericola@izs.it (M.S.); r.romantini@izs.it (R.R.); f.marotta@izs.it (F.M.); f.pomilio@izs.it (F.P.); f.demassis@izs.it (F.D.M.); 2 ASL Pescara, Unità Operativa Complessa Servizio di Sanità Animale, 65128 Pescara, Italy; andrea.diprovvido@asl.pe.it

**Keywords:** faeces, broilers, *Salmonella infantis*

## Abstract

Human salmonellosis incidence is increasing in the European Union (EU). *Salmonella* *enterica* subsp. *enterica* serovar Enteriditis, *Salmonella* *enterica* subsp. *enterica* serovar Typhimurium (including its monophasic variant) and *Salmonella* *enterica* subsp. *enterica* serovar Infantis represent targets in control programs due to their frequent association with human cases. This study aimed to detect the most prevalent serotypes circulating in Abruzzo and Molise Regions between 2015 and 2020 in the framework of the Italian National Control Program for Salmonellosis in Poultry (PNCS)]. A total of 332 flocks of Abruzzo and Molise Regions were sampled by veterinary services in the period considered, and 2791 samples were taken. Samples were represented by faeces and dust from different categories of poultry flocks: laying hens (*n* = 284), broilers (*n* = 998), breeding chickens (*n* = 1353) and breeding or fattening turkeys (*n* = 156). Breeding and fattening turkeys had the highest rate of samples positive for *Salmonella* spp. (52.6%; C.I. 44.8%–60.3%). Faeces recovered through boot socks represented the greatest number of positive samples (18.2%). *Salmonella* *enterica* subsp. *enterica* serovar Infantis was the prevalent serotype in breeding and fattening turkeys (32.7%; C.I. 25.8%–40.4%) and in broiler flocks (16.5%; C.I. 14.4%–19.0%). *Salmonella* *enterica* subsp. *enterica* serovar Typhimurium was detected at low levels in laying hens (0.7%; C.I. 0.2%–2.5%) followed by breeding and fattening turkeys (0.6%; C.I. 0.2%–2.5%). *Salmonella* *enterica* subsp. *enterica* serovar Enteriditis was also detected at low levels in laying hens (2.5%; C.I. 1.2%–5.0%). These findings highlight the role of broilers and breeding/fattening turkeys as reservoirs of *Salmonella* spp. and, as a consequence, in the diffusion of dangerous serotypes as *Salmonella* *enterica* subsp. *enterica* serovar Infantis. This information could help veterinary services to analyze local trends and to take decisions not only based on indications from national control programs, but also based on real situations at farms in their own competence areas.

## 1. Introduction

*Salmonella* spp. is the second most important etiological agent of gastrointestinal infection in humans after campylobacteriosis, and it is responsible for food-borne outbreaks in the EU/EEA, frequently associated with the consumption of poultry meat. *Salmonella* national control programs in poultry are implemented by EU Member States (MS) according to the EU Regulation (EC) No 2160/2003 and its amendments. As reported in the 2019 EU One Health Zoonoses Report, 70.0% of all serotyped *Salmonella* isolates, from food and animal sources, were from the broiler source. *Salmonella enterica* subsp. *enterica* serovar Infantis was the serotype most associated with broilers (93.1% of human cases of salmonellosis in the EU) and Italy was the MS who reported the highest percentage of isolates of this serotype (50.6%) [1].

The Italian National Health System has implemented control programs for *Salmonella* in poultry populations over the years (“Piano Nazionale di controllo delle salmonellosi negli avicoli, PNCS”) in order to reduce the prevalence of infection and to identify emerging serotypes of public concern through the introduction of sanitary measures against those that are considered as more dangerous for human health. The application of the PNCS is mandatory, throughout the national territory, for the following commercial poultry flocks: breeding chickens of the species *Gallus gallus,* laying hens of the species *Gallus gallus*, broilers of the species *Gallus gallus* and breeding and fattening turkeys of the species *Meleagris gallopavo. Salmonella enterica* subsp. *enterica serovar* Enteriditis, *Salmonella enterica* subsp. *enterica* serovar Typhimurium (including the monophasic variant with antigenic formula 1,4 [5], 12: i:-), *Salmonella enterica* subsp. *enterica* serovar Infantis, *Salmonella enterica subsp. enterica serovar* Virchow, *Salmonella enterica* subsp. *enterica* serovar Hadar for the breeding groups of *Gallus gallus,* and *Salmonella enterica* subsp. *enterica* serovar Enteriditis and *Salmonella enterica* subsp. *enterica* serovar Typhimurium (including the monophasic variant) for the groups of laying hens, broilers and breeding and fattening turkeys are the relevant serotypes of public concern considered in control programs. The PNCS control activities aim to achieve the objective of prevalence reduction of *Salmonella* serotypes to 1.0% or less for breeding, broilers of *Gallus gallus* and breeding and fattening turkeys’ flocks and to 2.0% or less for laying hens’ flocks. Since 2019, restrictive measures have been also implemented in case of identification of *Salmonella* *enterica* subsp. *enterica* serovar Infantis, an emerging serotype, in breeding chickens *Gallus gallus* [2]. Local veterinary systems, in case of detection of *Salmonella enterica* subsp. *enterica* serovar Enteriditis and/or *Salmonella enterica* subsp. *enterica* serovar Typhimurium, including the monophasic variant in a flock, must declare the group positive. Animals belonging to the group must be immediately subjected to health restrictions. Biosecurity measures must be reinforced at the farm level. Animals could be slaughtered and then destroyed in accordance with Regulation 1069/2009/EC or sent to the slaughterhouse, ensuring the separation between batches and the adoption of measures to ensure sanification procedures for environments and equipment. Groups of laying hens can be brought to the end of the cycle in compliance with the final destination of the eggs according to the Regulation 1237/2007/EC. Eggs from positive groups for *Salmonella enterica* subsp. *enterica* serovar Enteriditis or *Salmonella enterica* subsp. *enterica* serovar Typhimurium are not eligible for hatching. At the hatchery, eggs from positive flocks are destroyed and additional cleaning and disinfection of facilities and equipment must be carried out. Breeding flocks of *Gallus gallus,* positive for *Salmonella enterica* subsp. *enterica* serovar Infantis, are subjected to immediate health restrictions and all measures listed above must be applied. Eggs intended for consumption from laying hens’ flocks found positive for relevant serotypes, whose health status is unknown, or who are suspected of infection or recognized as source of infection in human food borne outbreaks are destroyed or identified as category B according to Regulation 589/2008/EC. In case of detection of *Salmonella enterica* subsp. *enterica* serovar Hadar and *Salmonella enterica* subsp. *enterica* serovar Virchow in breeding flocks of *Gallus gallus,* it is necessary to carry out an epidemiological investigation, in order to decide if the frequency of official controls on the farm should be intensified, and to request changes and/or supplementary actions to biosecurity measures. Cleaning, disinfection and pest control actions on the shed hosting the positive group must be carried out at the end of the cycle before the following restocking. The decontamination must be confirmed by a microbiological environmental control with at least five environmental swabs (sponge bags) carried out at least 10 days before the introduction of new groups, which can be authorized only after a negative result for the laboratory research of *Salmonella* spp. Vaccination against the serotype isolated in the previous cycle is mandatory only for breeding and laying hens used to repopulate a shed that housed, during the previous cycle, a group positive for *Salmonella enterica* subsp. *enterica* serovar Enteriditis and/or *Salmonella enterica* subsp. *enterica* serovar Typhimurium. The deeper analysis of data in specific areas of EU Member States is important not only to highlight different local trends able to confirm or not the EU trend, but also in the context of the identification of risk factors related to the spread of the infection, through the identification and characterization of circulating serotypes. This study, therefore, aimed to identify the most prevalent serotypes of *Salmonella* spp. circulating in two regions of Central Italy (Abruzzo and Molise Regions), between 2015 and 2020, in the framework of the “*Piano Nazionale di controllo delle salmonellosi negli avicoli*” (PNCS) [3,4,5].

## 2. Materials and Methods

### 2.1. Sampling

Samples collected by the veterinary services of Abruzzo and Molise Regions from 2015 to 2020 were tested in this study. The number of farms sampled every year was indicated in the PNCSs. The complete sampling protocol of PNCS was applied on farms with 250 animals or more, while a simplified protocol (adapted to each farm’s characteristics) of PNCS was used for smaller farms. Risk assessment procedures were applied on each farm to establish the sampling frequency and number of samples to collect each time. According to the PNCSs, flocks of caged and free-range laying hens (*Gallus gallus)*, free-range broilers (*Gallus gallus*), caged and free-range breeding chickens, caged and free-range breeding turkeys and free-range fattening turkeys (*Meleagris gallopavo*) were monitored during the study period. One or more than one category of flocks was tested at a single farm.

Two different matrices were sampled: faeces and dust. For caged flocks, two pools of fresh faeces (about 150 g each) were collected from the manure belt or from 60 different points of the manure pit, using disposable spatulas. For all the other flock categories, faeces were collected from the litter by walking through the flocks with boot cover swabs worn over the boots (boot swabs). At least two pairs of boot swabs were taken for sampling. Before putting the boot swabs on, their surface was moistened by the application of diluents approved by the national reference laboratory referred to in Article 11 of Regulation (EC) No 2160/2003. All sections in a house were represented in the sampling in a proportionate way. Each pair of boot swabs covered about 50% of the area of the house. On completion of sampling, the swabs were carefully removed from the boots so as not to dislodge adherent material. They were placed in a bag and labeled. Representative sampling was ensured basing on a case-by-case evaluation of epidemiological parameters, such as biosecurity conditions, the distribution or size of the flock by the competent authority.

Finally, for all flock categories, dust samples of about 100 g were collected from multiple places throughout the house from surfaces with visible presence of dust using disposable spatulas. As an alternative, one or several moistened fabric swabs of a combined surface of at least 900 cm^2^ were used to gather dust from multiple surfaces throughout the house. Each swab was coated with dust on both sides [6].

Samples were stored at room temperature and delivered within 24 h to the laboratory for bacteriological investigations. Isolation of *Salmonella* spp. was performed according to EN/ISO 6579-1:2017 [7]. Briefly, samples were pre-enriched in peptone buffered water (PBW) (Biolife Italiana, Milan, Italy). Aliquots of 25 g of fresh faeces or dust, individual gauze swabs, and the two or three pairs of boot socks per sample were each placed in 225 mL of PBW and incubated at 37 °C ± 1 °C for 18 ± 2 h. Pre-enriched cultures were inoculated onto modified semi-solid Rappaport-Vassiliadis agar plates by dispensing three drops (0.1 mL each) of pre-enriched broth, equally spaced on the medium surface. Appearance of a gray-white turbid zone (migration zone) surrounding the three drops was suggestive of bacterial motility and indicative of *Salmonella* growth. A loop of semi-solid agar from the furthest edge of the migration zone was sub-cultured on both Rambach agar and xylose lysine deoxycholate (XLD) agar. The presence of *Salmonella* spp. on Rambach agar and XLD agar was confirmed biochemically using the EnteroPluri-Test bacterial identification system (Liofilchem, Teramo, Italy), based on sugar fermentation.

At least one *Salmonella* spp. isolate was sub-cultured on triple sugar iron agar and subsequently serotyped according to ISO/TR 6579-3:2014 [8]. Serotyping was performed by slide agglutination with monovalent and polyvalent antisera (Statens Serum Institut, Copenhagen, Denmark), according to the Kauffmann–White–Le Minor scheme [9].

### 2.2. Statistical Analysis

To take into account the uncertainty of the proportion of positive laboratory results over the total tests performed, a beta distribution was used to define the 95% confidence interval of the proportion accuracy. The uncertainty interval was defined as the difference between upper and lower 95% confidence limits. The 95% lower and upper credibility levels (L.C.I. and U.C.L., respectively, composing the Credibility Interval, C.I.) of the distribution frequency of positive results were calculated using a Bayesian approach with a beta distribution β(n + 1; *n* − s + 1), where n is the total number of tested samples and are the tested positive samples [10].

## 3. Results

Based on PNCS, a total of 332 flocks were tested in Abruzzo and Molise Regions: 54 flocks of laying hens (16.3%), 209 of broilers (63.0%), 58 of breeding chickens (17.5%) and 11 of breeding and fattening turkeys (3.3%). Laying hen flocks were prevalent in Pescara Province (20). Broiler flocks were mostly found in Campobasso Province (153). Breeding chicken flocks were present at similar levels in Teramo, Campobasso and Isernia provinces (17, 16 and 14 flocks, respectively) while turkey flocks (breeding and fattening) were mainly found in Teramo Province (10). Overall, 53.6% of tested flocks (178) were located in Campobasso Province. For each province the total number of flocks (332) and farms (304) considered over the years is different. This is due to the fact that each farm could incorporate more than one type of flock (Table 1).

Teramo and Campobasso were, also, the provinces with the highest number of farms tested over the years, with 35 and 173 farms, respectively.

**Figure 1:**
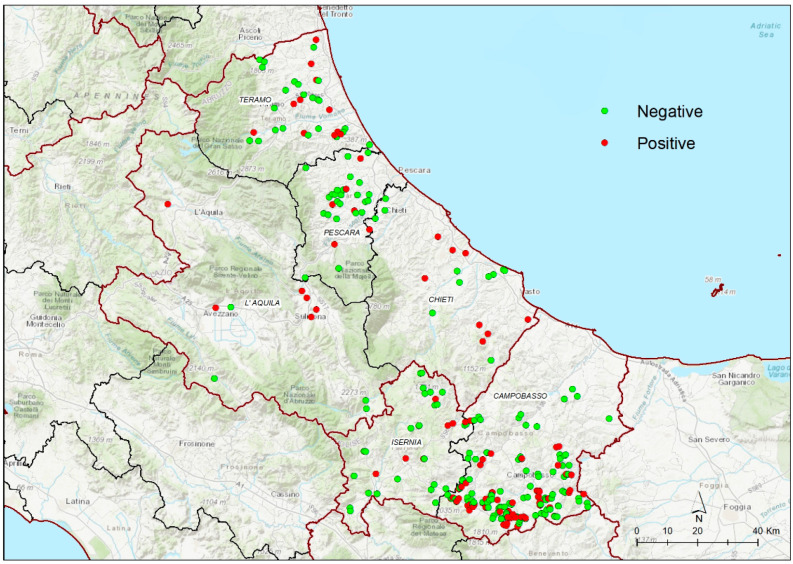
Map of central Italy showing locations of poultry farms tested for *Salmonella* spp. in Abruzzo and Molise Regions between 2015 and 2020. Red dots represent farms where *Salmonella* spp. was detected at least once over the study period; green dots represent farms where *Salmonella* spp. was never detected. Each farm could incorporate more than one type of flock.

*Salmonella* spp. was detected at least once in 14 (28.6%) farms in Teramo Province and in 66 (37.1%) farms in Campobasso Province. In Isernia Province, 38 farms were tested with seven (18.4%) farms positive at least once in the period considered. Positive farms in Pescara Province were six out of the 29 farms tested (20.7%). In Chieti Province 19 farms were tested with 12 (63.2%) testing positive at least once. Finally, in L’Aquila Province, 10 farms were tested with six (60.0%) positive farms found at least once over the years. The geographical distribution of farms considered in the study is shown in Figure 1.

Overall, 2791 samples were collected from 2015 to 2020. *Salmonella* spp. was detected in 390 samples (14.0%) (Table 2). A peak of 93 positive samples out of 485 tested (19.2%) was registered in 2018.

**Figure 2:**
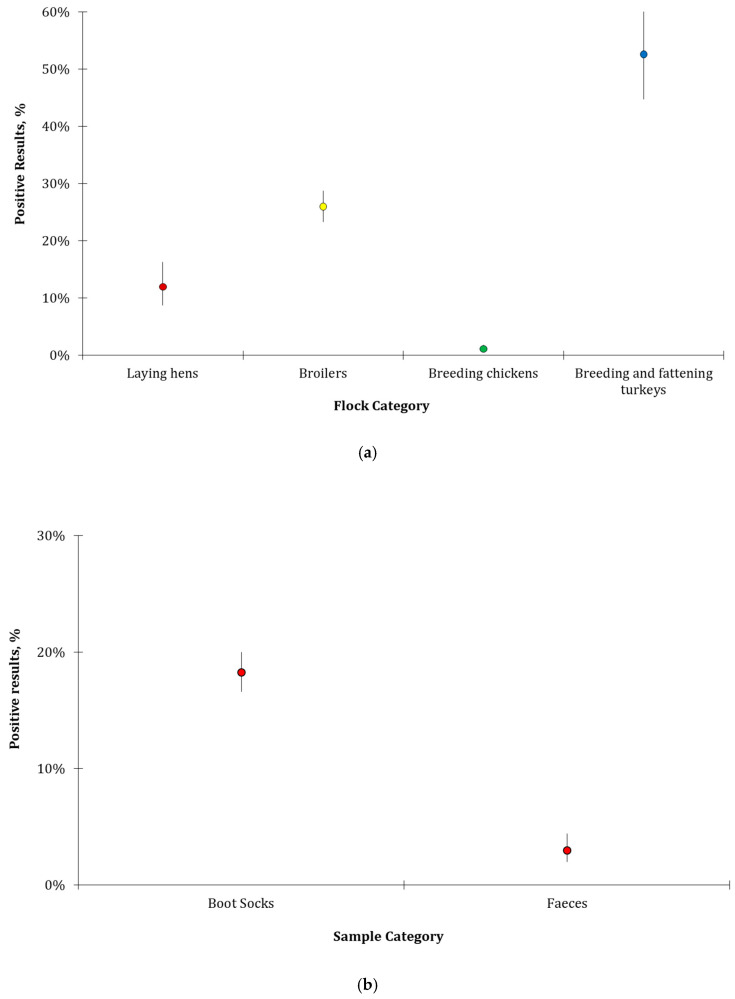

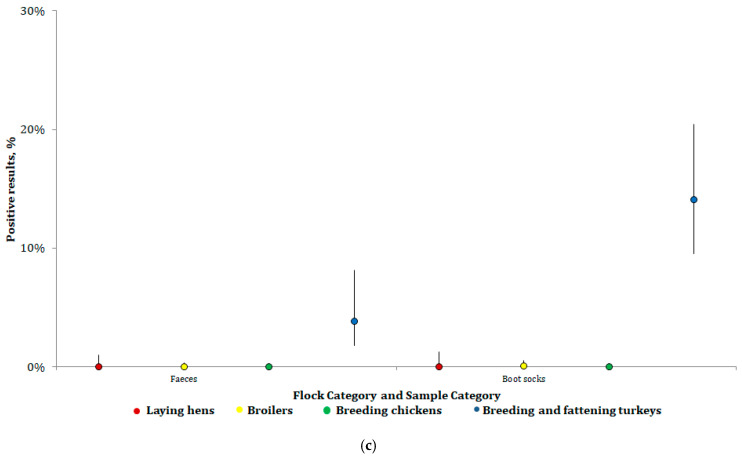
Percentages of samples positive to *Salmonella* spp. collected between 2015 and 2020 in Abruzzo and Molise Regions and related C. I. (**a**) Percentages of positive samples per flock category; (**b**) Percentages of positive samples per sampling method; (**c**) Percentages of positive samples according to sampling method and flock categories.

During the six-year period under study, the percentage of isolation was higher in breeding and fattening turkeys than in other categories (52.6%; C.I. 44.8%–60.3%) (Figure 2a, Table 3. Most of the positive results were collected from faeces taken through boot socks (18.2%; C.I. 16.6%–20.0%) while only a few positive results were obtained from fresh faeces (3.0%; C.I. 2.0%–4.4%) (Figure 2b, Table 4). Faeces from breeding and fattening turkeys, recovered through boot socks over all the years, represented the 14.1% (C.I. 9.5%–20.4%) of positive samples while samples of fresh faeces from the same category represented the 3.8% (C.I. 1.8%–8.1%) of positive samples. (Figure 2c, Table 5). The dust samples collected were six, and all tested negative to *Salmonella* spp. For these reasons, dust samples have not been considered in specific calculations.

*Salmonella* spp. isolates were serotyped to investigate the serotypes of public concern, and 393 serotypes were identified. In particular, two serotypes were isolated in a boot sock sample and three serotypes in another boot sock. *Salmonella* *enterica* subsp. *enterica* serovar Infantis was the most prevalent serotype, with 225 isolates (57.3%) found among the 393 strains in the period from 2015 to 2020 in both regions (Table 6).

The study also highlighted the presence of other serotypes of *Salmonella* spp. circulating in both regions. *Salmonella* *enterica* subsp. *enterica* serovar Livingstone was the second-most detected serotype overall (67 isolates, 17.0% of the total), followed by *Salmonella* *enterica* subsp. *enterica* serovar Derby (23 isolates, 5.9%).

The provincial distribution of the number of serotypes ranged widely. A total of 142 and 116 serotyped isolates (isolates) came from Teramo and Campobasso Provinces, respectively, according to the number of farms considered and that tested positive over the years. Instead 104 isolates were from Chieti Province. In this case the number of farms tested over the years (19) was lower than other provinces, but the percentages (63.1%) of farms tested positive (12) over the years was higher. This was due to the fact that 7 of 12 farms tested positive from three to six times (at least one time per year considered in the study). In L’Aquila Province only two of six farms tested positive from three to six times (at least one time per year). Here the number of isolates was low (only 11). A total of 19 isolates came from Pescara Province. In this case the percentages of farms tested positive was 20.6% (six farms). Only from one farm were positive samples obtained over three consecutive years, during the period under study. Regarding Isernia Province, 10 positive samples were obtained from seven farms tested positive, but only one isolate was serotyped.

**Figure 3:**
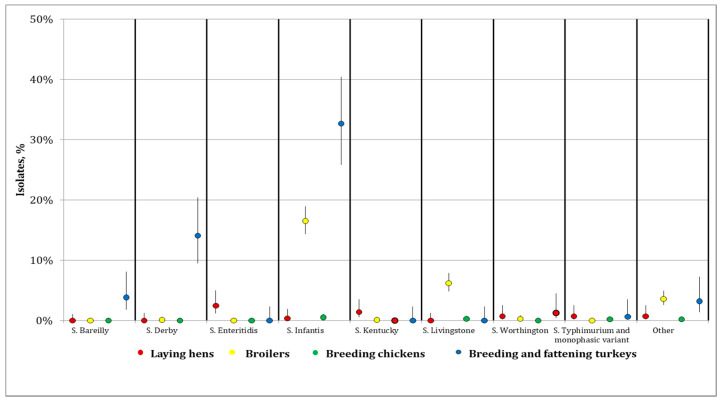
Distribution of *Salmonella* spp. serotypes by flock category.

Finally, the distribution of positive samples was investigated in the different flock categories. *Salmonella* *enterica* subsp. *enterica* serovar Infantis was mostly isolated in breeding and fattening turkeys (32.7%; C.I. 25.8%–40.4%), which was the category that showed the highest percentage of isolation, followed by broilers (16.5%; C.I. 14.4%–19.0%) (Figure 3). *Salmonella* *enterica* subsp. *enterica* serovar Derby and *Salmonella* *enterica* subsp. *enterica* serovar Bareilly were isolated mainly from breeding and fattening turkeys (14.1%; C.I. 9.5%–20.4% and 3.8%; C.I. 1.8%–8.1%, respectively). *Salmonella* *enterica* subsp. *enterica* serovar Livingstone was prevalent among broilers (6.2%; C.I. 4.9%–7.9%). Two strains of public health concern, namely *S*.*Salmonella* *enterica* subsp. *enterica* serovar Typhimurium and *Salmonella* *enterica* subsp. *enterica* serovar Enteriditis, were detected at low levels. *Salmonella* *enterica* subsp. *enterica* serovar Enteriditis was detected only from laying hens with a percentage of isolation of 2.5%; C.I. 1.2%–5.0%.

## 4. Discussion

This study considered samples taken in the framework of national control programs (PNCSs) in Abruzzo and Molise Regions between 2015 and 2020. Samples of faeces and dust came from different types of flocks: laying hens, broilers, breeding chickens, and breeding and fattening turkeys. During the period under study, breeding and fattening turkeys was the category where the percentage of positive results was the highest, followed by broilers.

The trend of detection increased from 2015 to 2018 (when 19.2% of samples tested positive), then it decreased till 2020 (when 9.0% samples tested positive). In 2019, as reported in the EU One Health Zoonoses Report 2019, Italy met the target of 1% or less of broiler flocks of *Gallus gallus* positive for *Salmonella* *enterica* subsp. *enterica* serovar Enteriditis and/or *Salmonella* *enterica* subsp. *enterica* serovar Typhimurium (including monophasic variants). In our study, according to expectations, *Salmonella* *enterica* subsp. *enterica* serovar Enteriditis and *Salmonella* *enterica* subsp. *enterica* serovar Typhimurium were detected at low levels with only 14 isolates in all categories during the period under study.

According to EU Regulation (EC) No 2160/2003, Italy had to set up national control programs (PNCSs) in order to reduce the prevalence of *Salmonella* serovars (targets) relevant for public health. *Salmonella* *enterica* subsp. *enterica* serovar Enteriditis and *Salmonella* *enterica* subsp. *enterica* serovar Typhimurium (including monophasic variant) were indicated as target serovars for laying hens, broilers, breeding and fattening turkeys. *Salmonella* *enterica* subsp. *enterica* serovar Enteriditis, *Salmonella* *enterica* subsp. *enterica* serovar Typhimurium (including monophasic variant), *Salmonella* *enterica* subsp. *enterica* serovar Infantis, *Salmonella* *enterica* subsp. *enterica* serovar Virchow and *Salmonella* *enterica* subsp. *enterica* serovar Hadar were targets for breeding hens. In our study the most prevalent serotype was represented by *Salmonella* *enterica* subsp. *enterica* serovar Infantis (32.7%; C.I. 25.8%–40.4%) followed by *Salmonella* *enterica* subsp. *enterica* serovar Derby. Although *Salmonella* *enterica* subsp. *enterica* serovar Infantis is not considered a target serotype for breeding/fattening turkeys and broilers, it is the most frequently isolated in broiler flocks and the fourth most common in breeding flocks and laying hens in the EU [11]. *Salmonella* *enterica* subsp. *enterica* serovar Infantis is a serotype of public health concern due to its frequent isolation from humans. In this serotype the presence of multidrug resistance patterns is also frequent. Proietti et al. (2020) found a high number of multi-resistant strains (98.0%) isolated from the food chain of broiler meat production. Resistance to cephalosporines is a problem of big concern for public health because this type of antibiotic is considered one of the last choices in human medicine [12]. Moreover, it has been shown that production of fimbriae and cellulose and the ability to form biofilm are important factors for the survival of *Salmonella* on surfaces and its persistence in the environment, especially in sites full of protective organic materials. In contrast, a recent study demonstrated that the persistence of *Salmonella* *enterica* subsp. *enterica* serovar Infantis on broiler farms could be more related to ineffective disinfection protocols than to its ability to form biofilm [13].

The serovars isolated in our study from laying hens were *Salmonella* *enterica* subsp. *enterica* serovar Enteriditis, *Salmonella* *enterica* subsp. *enterica* serovar Kentucky, *Salmonella* *enterica* subsp. *enterica* serovar Typhimurium and monophasic variant, and *Salmonella* *enterica* subsp. *enterica* serovar Worthington. *Salmonella* *enterica* subsp. *enterica* serovar Enteriditis is able to colonize reproductive tissues in infected hens and, consequently, the edible content of eggs, so it represents an international public health concern. In addition, high poultry-stocking densities may cause stress, which could suppress the immune responses and facilitate *Salmonella* invasion of internal organs [14]. Airborne transmission of *S.* Enteriditis is an important element that influences the spreading of infections between cages of laying hens. Mollenhorst et al. (2005) showed that the effect of flock size was important, and that bigger flocks had a higher chance of infection with *Salmonella* *enterica* subsp. *enterica* serovar Enteriditis in all housing systems [15]. *Salmonella* *enterica* subsp. *enterica* serovar Kentucky is considered a high priority resistant pathogen, and, according to the WHO, already in the global priority list [16]. It has been identified as the most frequently isolated *Salmonella* serovar from broiler chickens in USA from 1998 to 2013, but this trend is not consistent on a global scale [17].

*Salmonellaenterica* subsp. *enterica* serovar Infantis, in our study, was the prevalent serotype among breeding and fattening turkeys, where it was followed by *Salmonella enterica* subsp. *enterica* serovar Derby in terms of positive samples collected. This serovar is emerging in Europe as a predominant serovar in fattening turkey flocks and it was found to be predominant in turkeys in 2014 in United Kingdom (UK). In 2016 *Salmonella enterica* subsp. *enterica* serovar Derby was also detected in turkeys and broilers in Ireland and Spain [18].

In our study, faeces collected through boot socks or taken directly from the litter were used to test the presence of *Salmonella* spp. It has previously been reported that sampling fecal material in a *Salmonella*-infected chicken flock by means of two pairs of boot socks, both analyzed as one sample, was at least as effective in detecting Salmonella as hand collection of 60 faeces, analyzed as one sample [19]. However, this is in contrast with the results of our study, where the probability of isolating Salmonella by using boot socks is significantly higher than the probability of isolation through the hand-collection of faeces. The largest number of positive samples was collected using boot socks. Since the 1980s, the presence of *Salmonella* spp. in poultry flocks has been determined through litter sampling because it is a non-invasive, cost effective and practical way for the detection of *Salmonella* spp. [20]. In a previous study, the use of boot covers soaked in saline showed higher detection than drag swab sampling, litter grab sampling and fecal samples [21]. Boot swabs allows collection of pooled faeces from floor housing systems. It is preferable to collect naturally pooled faeces instead of collecting individual cloacal swabs. It is also important to avoid contamination or contact with disinfectant prior to using boot socks [22].

Dust is regarded also as a sensitive sample type for detecting Salmonella and it is also a potential vehicle of transmission of Salmonella in some types of poultry houses [23]. In our study we did not obtain positive samples from dust. Actually, samples collected were only six, and probably this is due to the difficulties in sampling dust when horizontal surfaces, where the dust can settle, are not available or when the presence of naturally ventilated houses hampers sedimentation of dust. Dust accumulates especially around fans and extraction fans where it is not so easy to sample.

This study highlights the occurrence of *Salmonella* spp. relevant serotypes in farms located in Abruzzo and Molise Regions. *Salmonella* *enterica* subsp. *enterica* serovar Enteriditis and *Salmonella* *enterica* subsp. *enterica* serovar Typhimurium (and its monophasic variant) were detected at low levels, and this finding is in accordance with the target set up for MS on prevalence reduction of both serotypes of 1% for breeding chickens *Gallus gallus* and 2% for laying hens. *Salmonella* *enterica* subsp. *enterica* serovar Infantis, instead, was the prevalent serotype among breeding and fattening turkeys and broilers. Both categories are not included in the PNCS among those interested by sanitary measures, which are the breeding chickens *Gallus gallus*. Extended studies of prevalence are needed in order to verify if this result is due to a local geographical trend or if it is related to a national trend; this is in light of the need to integrate recommendations in the PNCS if necessary. Further research is needed to evaluate patterns of antimicrobial resistance among isolates, especially among those of *Salmonella* *enterica* subsp. *enterica* serovar Infantis and to detect clones circulating in the area under study. This could be done through the application of next generation sequencing techniques.

## Figures and Tables

**Table 1:** Flocks tested in the period 2015–2020 in Abruzzo and Molise provinces, by category. Values are numbers of flocks.

Province	Laying Hens (*n*)	Broilers (*n*)	Breeding Chickens (*n*)	Breeding and Fattening Turkeys (*n*)	Total (*n*)
TE	4	18	17	10	49
AQ	10	0	1	0	11
PE	20	7	7	0	34
CH	8	9	3	1	21
CB	9	153	16	0	178
IS	3	22	14	0	39
Total	54	209	58	11	332

CH: Chieti; AQ: L’Aquila; PE: Pescara; TE: Teramo; IS: Isernia; CB: Campobasso.

**Table 2:** Yearly percentages of samples resulted positive to isolation of *Salmonella* spp.

Year	Total (*n*)	Positive (*n*)	Percentage of Positive Samples
2015	495	58	11.7 %
2016	604	78	12.9 %
2017	483	81	16.8 %
2018	485	93	19.2 %
2019	359	47	13.1 %
2020	365	33	9.0 %
Total	2791	390	14.0 %

**Table 3:** Percentages of samples positive to *Salmonella* spp. collected between 2015 and 2020 in Abruzzo and Molise Regionsby flock category.

	Laying Hens	Broilers	Breeding Chickens	Breeding and Fattening Turkeys
Tested	284	998	1353	156
Negative	250	739	1338	74
Positive	34	259	15	82
U.C.L. 95%	16.3%	28.8%	1.8%	60.3%
L.C.L. 95%	8.7%	23.3%	0.7%	44.8%
Positive (%)	12.0%	26.0%	1.1%	52.6%

**Table 4:** Percentages of positive results, according to the sampling method.

	Boot Socks	Faeces
Tested	2011	774
Negative	1644	751
Positive	367	23
U.C.L. 95%	20.0%	4.4%
L.C.L. 95%	16.6%	2.0%
Positive (%)	18.2%	3.0%

**Table 5:** Percentages of positive results, according to the matrix associated with its sampling method by flock category.

		Faeces				Boot Socks		
	Laying Hens	Broilers	Breeding Chickens	Breeding and Fattening Turkeys	Laying Hens	Broilers	Breeding Chickens	Breeding and Fattening Turkeys
Tested	284	998	1353	156	284	998	1353	156
Negative	284	0	0	0	0	0	0	0
Positive	0	0	0	6	0	1	0	22
U.C.L. 95%	1.0%	0.4%	0.3%	8.1%	1.3%	0.6%	0.3%	20.4%
L.C.L. 95%	0.0%	0.0%	0.0%	1.8%	0.0%	0.0%	0.0%	9.5%
Positive (%)	0.0%	0.0%	0.0%	3.8%	0.0%	0.1%	0.0%	14.1%

**Table 6:** *Salmonella* spp. serotypes isolated in individual provinces of Abruzzo and Molise Regions between 2015 and 2020. Values are numbers of isolates.

Serotype	Abruzzo				Molise		Total, *n* (%)
	CH	AQ	PE	TE	IS	CB	
*Salmonella enterica* subsp. *enterica* serovar Bareilly	0	0	0	6	0	0	6 (1.5)
*Salmonella enterica* subsp. *enterica* serovar Derby	22	0	0	0	0	1	23 (5.9)
*Salmonella enterica* subsp. *enterica* serovar Enteritidis	0	4	0	1	0	2	7 (1.8)
*Salmonella enterica* subsp. *enterica* serovar Infantis	52	0	6	94	0	73	225 (57.3)
*Salmonella enterica* subsp. *enterica* serovar Kentucky	0	4	1	0	0	0	5 (1.3)
*Salmonella enterica* subsp. *enterica* serovar Livingstone	19	0	8	23	0	17	67 (17.0)
*Salmonella enterica* subsp. *enterica* serovar Worthington	0	2	2	2	0	1	7 (1.8)
*Salmonella enterica* subsp. *enterica* serovar Typhimurium and monophasic variant	1	0	1	5	0	0	7 (1.8)
Other *	10	1	1	11	1	22	46 (11.7)
Total	104	11	19	142	1	116	393 (100)

CH: Chieti; AQ: L’Aquila; PE: Pescara; TE: Teramo; IS: Isernia; CB: Campobasso.* Other: 32 *Salmonella* spp. serotypes each detected from one to three times over the period considered.

## Data Availability

All raw data are available in a proprietary repository at the Istituto Zooprofilattico Sperimentale dell’Abruzzo e del Molise “G. Caporale” and can be made available upon request.
